# Supplementary material for: Pupil-linked phasic arousal predicts a reduction of choice bias across species and decision domains
Source: eLife. 2020 Jun 16;9:e54014. doi: 10.7554/eLife.54014 (PMC7297536; doi:10.7554/eLife.54014)
Supplement: Transparent reporting form [file elife-54014-transrepform.docx]

***eLife’s* transparent reporting form**

We encourage authors to provide detailed information *within their submission* to facilitate the interpretation and replication of experiments. Authors can upload supporting documentation to indicate the use of appropriate reporting guidelines for health-related research (see [EQUATOR Network](http://www.equator-network.org/%20)), life science research (see the [BioSharing Information Resource](https://biosharing.org/)), or the [ARRIVE guidelines](http://www.plosbiology.org/article/info:doi/10.1371/journal.pbio.1000412) for reporting work involving animal research. Where applicable, authors should refer to any relevant reporting standards documents in this form.

If you have any questions, please consult our Journal Policies and/or contact us: [editorial@elifesciences.org](mailto:editorial@elifesciences.org).

**Sample-size estimation**

- You should state whether an appropriate sample size was computed when the study was being designed
- You should state the statistical method of sample size computation and any required assumptions
- If no explicit power analysis was used, you should describe how you decided what sample (replicate) size (number) to use

Please outline where this information can be found within the submission (e.g., sections or figure legends), or explain why this information doesn’t apply to your submission:

In Material and Methods, section *Subjects* we write (p. 15):

“We analyzed two previously unpublished human data sets, and we re-analyzed a previously published mice data set (McGinley, David, et al., 2015) and two human data sets (Bergt, Urai, Donner, & Schwabe, 2018; de Gee et al., 2017). We selected the data set by Bergt *et al* (2018) for across-subject correlations of variables of interest (Fig. 4). This data set had a sufficient sample size for such an analysis, based on the effect size obtained in a previous study (de Gee *et al,* PNAS, 2014). The sample sizes (and trial numbers per individual) for the newly collected human data sets were determined based on the effects observed in a previous study comparing diffusion model parameters between pupil conditions: de Gee *et al.,* eLife*, 2017*, with N=14.”

**Replicates**

- You should report how often each experiment was performed
- You should include a definition of biological versus technical replication
- The data obtained should be provided and sufficient information should be provided to indicate the number of independent biological and/or technical replicates
- If you encountered any outliers, you should describe how these were handled
- Criteria for exclusion/inclusion of data should be clearly stated
- High-throughput sequence data should be uploaded before submission, with a private link for reviewers provided (these are available from both GEO and ArrayExpress)

Please outline where this information can be found within the submission (e.g., sections or figure legends), or explain why this information doesn’t apply to your submission:

Our manuscript describes a mouse and human pupillometry and behavior, and “biological” versus “technical” replication does not apply.

We did not exclude outliers.

Details on number of subjects and ranges of trial counts per subject are explicitly described in the sections *Subjects* and *Behavioral tasks*, respectively (Materials and Methods).

**Statistical reporting**

- Statistical analysis methods should be described and justified
- Raw data should be presented in figures whenever informative to do so (typically when N per group is less than 10)
- For each experiment, you should identify the statistical tests used, exact values of N, definitions of center, methods of multiple test correction, and dispersion and precision measures (e.g., mean, median, SD, SEM, confidence intervals; and, for the major substantive results, a measure of effect size (e.g., Pearson's r, Cohen's d)
- Report exact p-values wherever possible alongside the summary statistics and 95% confidence intervals. These should be reported for all key questions and not only when the p-value is less than 0.05.

Please outline where this information can be found within the submission (e.g., sections or figure legends), or explain why this information doesn’t apply to your submission:

Statistical analysis methods are described in detail in the Materials and Methods section *Statistical comparisons* (p. 23-24). We used mixed-linear modeling procedures or paired-samples t-tests throughout, and present exact p-values all throughout (see Figures). Furthermore, each Figure and or Figure legend includes information on the statistical tests used, exact values of N, definitions of center, methods of multiple test correction, and dispersion and precision measures.

(For large datasets, or papers with a very large number of statistical tests, you may upload a single table file with tests, Ns, etc., with reference to sections in the manuscript.)

**Group allocation**

- Indicate how samples were allocated into experimental groups (in the case of clinical studies, please specify allocation to treatment method); if randomization was used, please also state if restricted randomization was applied
- Indicate if masking was used during group allocation, data collection and/or data analysis

Please outline where this information can be found within the submission (e.g., sections or figure legends), or explain why this information doesn’t apply to your submission:

We performed all analyses within subjects, and did not allocate subjects into groups.

**Additional data files (“source data”)**

- We encourage you to upload relevant additional data files, such as numerical data that are represented as a graph in a figure, or as a summary table
- Where provided, these should be in the most useful format, and they can be uploaded as “Source data” files linked to a main figure or table
- Include model definition files including the full list of parameters used
- Include code used for data analysis (e.g., R, MatLab)
- Avoid stating that data files are “available upon request”

Please indicate the figures or tables for which source data files have been provided:

We have provided csv-tables containing the first-level measures that served as input to each of the main figures 1-5.
